# The N-Terminal Region of the PA Subunit of the RNA Polymerase of Influenza A/HongKong/156/97 (H5N1) Influences Promoter Binding

**DOI:** 10.1371/journal.pone.0005473

**Published:** 2009-05-07

**Authors:** Takahito Kashiwagi, Bo Wah Leung, Tao Deng, Hualan Chen, George G. Brownlee

**Affiliations:** 1 Sir William Dunn School of Pathology, University of Oxford, Oxford, United Kingdom; 2 Division of Infectious Diseases, Department of Infectious Medicine, Kurume University School of Medicine, Fukuoka, Japan; 3 Department of Microbiology, The Chinese University of Hong Kong, Prince of Wales Hospital, Hong Kong SAR, China; 4 Division of Virology, National Institute of Biological Standards and Control, South Mimms, United Kingdom; 5 Harbin Veterinary Research Institute, Chinese Academy of Agricultural Sciences, Harbin, People's Republic of China; University of Hong Kong, Hong Kong

## Abstract

**Background:**

The RNA polymerase of influenza virus is a heterotrimeric complex of PB1, PB2 and PA subunits which cooperate in the transcription and replication of the viral genome. Previous research has shown that the N-terminal region of the PA subunit of influenza A/WSN/33 (H1N1) virus is involved in promoter binding.

**Methodology/Principal Findings:**

Here we extend our studies of the influenza RNA polymerase to that of influenza strains A/HongKong/156/97 (H5N1) and A/Vietnam/1194/04 (H5N1). Both H5N1 strains, originally isolated from patients in 1997 and 2004, showed significantly higher polymerase activity compared with two classical human strains, A/WSN/33 (H1N1) and A/NT/60/68 (H3N2) in vitro. This increased polymerase activity correlated with enhanced promoter binding. The N-terminal region of the PA subunit was the major determinant of this enhanced promoter activity.

**Conclusions/Significance:**

Overall we suggest that the N-terminal region of the PA subunit of two recent H5N1 strains can influence promoter binding and we speculate this may be a factor in their virulence.

## Introduction

Influenza A virus is a single-stranded, negative-sense RNA virus with an 8 segmented genome, belonging to the family Orthomyxoviridae [1]. The segmented genome is transcribed and replicated by a viral RNA-dependent RNA polymerase in infected cells [2]. Transcription requires a capped RNA primer which is “snatched” from RNA polymerase II transcripts in the host cell [3]–[4]. This cap snatching is performed by a cap-dependent endonuclease activity of the influenza RNA polymerase complex, generating short, 9 to 17-nucleotide, capped RNA primers [5]–[7]. On the other hand, replication is independent of a primer. A full-length positive sense RNA (cRNA) is initially synthesized from viral RNA (vRNA), and subsequently serves as a template for the synthesis of full-length negative-sense vRNA [1]–[2], [8].

The influenza virus RNA polymerase is a trimeric complex comprising three different subunits - PB1, PB2 and PA [1]–[2]. Electron microscopy shows that it is very compact with no apparent boundaries [9]–[11]. No high resolution structure of the trimeric polymerase complex is available yet, although amino acids 1–197 of the N-terminal region of PA subunit [13], [14], amino acids 257–716 of PA complexed with a short peptide at the N-terminus of PB1 [12] and short regions of the structure of PB2 [15]–[16] are known. All three subunits are generally found to be required for both transcription and replication [1], [17], although other reports disagree [18]. The PB1 subunit contains an SDD motif which is directly involved in RNA chain elongation [2], [19]–[20]. The PB2 subunit is involved in cap-snatching and cap-binding [1]–[2], [21]. The PA subunit is involved in transcription and replication as well as endonuclease activity, cap binding and promoter binding [6], [15], [17], [22]–[23]. Furthermore, the PA is known to have, or induce, proteolytic activity [24]–[26], although the significance of this function is not fully understood.

Influenza viruses of H5N1 subtype have been isolated in the past from chickens [27] and turkeys [28] (e.g. A/chicken/Scotland/59; A/turkey/England/91) but the current H5N1 isolates are believed to derive from geese in Guangdong Province, China in 1996 [29]. They received little attention until virus spread to humans in Hong Kong in May 1997, killing 6 of 18 infected people [30]–[33]. Since then this highly pathogenic avian influenza virus of the H5N1 strain has been circulating worldwide especially in Southeast Asia [34]–[36]. Although sporadic transmission from poultry to humans occasionally occurs, current avian H5N1 strains are not adapted to efficient human-human transmission [37]. It has been shown that avian-to-human transmission is limited by the receptor binding properties of the haemagglutinin of avian viruses [38]–[39] and by residue 627 of the PB2 subunit [40]–[41]. Nevertheless, the mechanism of transmission from birds to human is not fully understood. In most cases, H5N1 subtypes are highly pathogenic in humans and cause death (mortality ∼60%) or severe disease compared to classical human strains such as H3N2 and H1N1 [34], [42]. Recently, the contribution of RNA polymerase and nucleoprotein to pathogenesis of avian H5N1 influenza virus in chickens has been reported [43]–[44]. However, the molecular mechanism of the pathogenicity in chickens and in humans is still poorly understood.

In recent reports on the mechanism of genome replication, it has been shown that point mutants in the N-terminal region of the PA subunit of influenza A/WSN/33 virus can interfere with cRNA promoter binding, suggesting that this region of PA is involved, directly or indirectly, in regulating promoter binding [6], [22]–[23]. In order to extend these studies to more recently isolated strains, the RNA polymerase of A/Hong Kong/156/97 (H5N1) and A/Vietnam/1194/04 (H5N1) were compared here with one another and with the older, more classical strains of different subtypes, i.e. A/WSN/33 (H1N1) and A/NT/60/68 (H3N2). We found that the PA subunit of both of the polymerases of H5N1 subtype can substantially increase RNA polymerase activity by enhancing promoter binding in vitro. Analysis of chimeras of the PA subunit of A/HongKong/156/97 (H5N1) with A/WSN/33 (H1N1) showed that N-terminal domain of PA of A/HongKong/156/97 (H5N1) increased polymerase activity in vitro. However, unexpectedly, this N-terminal region of the PA of A/HongKong/156/97 (H5N1), when expressed as a ribonucleoprotein hybrid with the PB1, PB2 polymerase and NP subunits of A/WSN/33 virus, decreased transcription and/or replication in two different, cell-based assays in vivo, presumably by inhibiting promoter clearance in the initial stages of replication and/or replication. Our data highlight the importance of the N-terminal region of PA of more recent H5N1 strains in influencing viral replication.

## Results

### Comparison of polymerase activity of H5N1 human strains with classical H1N1 or H3N2 strains in vitro

**Figure 1:**
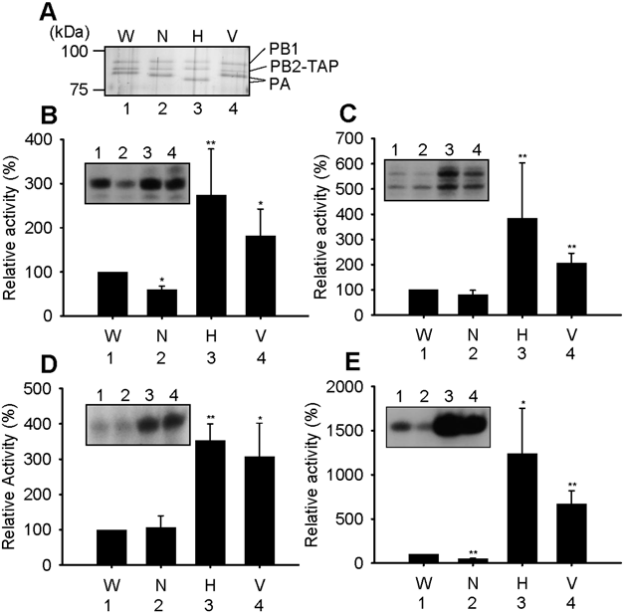
Comparison of polymerase activities of two H5N1 strains and two classical human strains in vitro. (A) 7.5% SDS-PAGE of WSN (W), NT (N), HK (H) and VN (V) partially purified polymerases analysed by silver staining. The positions of PB1, PB2-TAP and the variable positions of PA are indicated. (B) ApG-primed transcription of W, N, H & V polymerases (see Materials and Methods). (C) Globin mRNA-primed transcription of W, N, H & V polymerases (see Materials and Methods). (D) ApG synthesis with a model vRNA promoter and (E) with a model cRNA promoter of W, N, H & V polymerases (see Materials and Methods). Relative activity is % activity relative to WSN (W) from at least 3 independent experiments. Inset square panels in B–E show typical results. * and ** show statistical significance at p<0.05 and p<0.01, respectively, in a Student's t-test.

In order to test if the RNA polymerases of human-isolated H5N1 strains differed from the polymerase of classical strains, we initially compared recombinant polymerase activities of 2 human-isolated avian strains A/HongKong/156/97 (H5N1) [HK] and A/Vietnam/1194/04 (H5N1) [VN] with 2 classical human strains of different subtypes A/WSN/33 (H1N1) [WSN] and A/NT/60/68 (H3N2) [NT] in vitro. Figure 1A shows the trimeric complex of influenza polymerase, which was transiently expressed in human 293T cells and partially purified utilizing a TAP (tandem affinity purification) tag on the C-terminus of the PB2 subunit (see Materials and Methods). The migration of PB1 and PB2 subunits were identical in each strain (Figure 1A). However, the PA subunit migrated differently, as observed before [23]. After quantitation of the yields of the PA subunits (see Materials and Methods), polymerase preparations were normalized to a standard amount of polymerase (Figure 1A) before testing enzymatic activity.

Initially, we performed an ApG-primed transcription assay [45] and a globin mRNA-primed transcription assay [18] with a short model vRNA promoter (see Materials and Methods). In both assays (Figure 1B and C), both HK and VN H5N1 strains showed significantly higher activity, ranging from 2 to 4 fold, than the classical human strains (WSN and NT) tested. To confirm the differences in polymerase activity observed with ApG and globin mRNA primers, we subsequently performed an ApG synthesis assay – a measure of replication initiation, using either a vRNA (Figure 1D) or a cRNA (Figure 1E) promoter as a short model template (see Materials and Methods). The two H5N1 strains (Figure 1D and E) showed significantly higher activity (3–11 fold) than the classical strains (Figure 1D and E, lanes 1 and 2), especially with the model cRNA promoter (Figure 1E). Overall, the four in vitro assays (Figure 1B–E) consistently showed that both H5N1 strains isolated from humans demonstrated significantly higher activities in model replication and transcription assays in vitro than those of classical human strains.

### Analysis of the contribution of the polymerase subunit to polymerase activity in vitro

**Figure 2 pone-0005473-g002:**
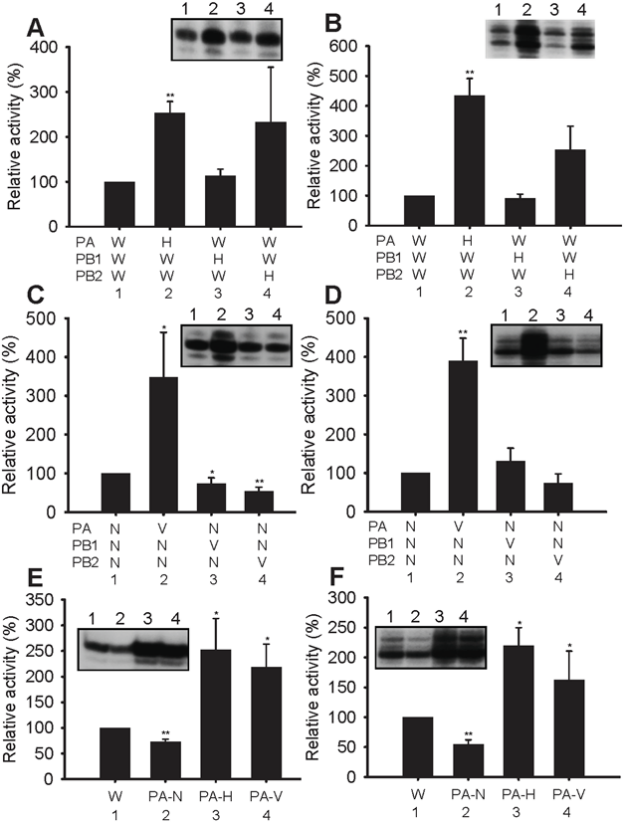
Comparison of activities of polymerase hybrids between H5N1 strains and classical human strains in vitro. (A) and (B) a WSN (W) subunit was replaced with the corresponding HK (H) subunit as indicated in 1–4. (C) and (D) a NT (N) subunit was replaced with the corresponding VN (V) subunit as indicated in 1–4. (E) and (F) The PA subunit of WSN (W) was replaced with either the PA of NT (PA-N), or HK (PA-H) or VN (PA-V). The activity was analyzed by the ApG-primed transcription assay (in A, C and E) and by the globin mRNA-primed transcription assay (in B, D and F). Relative activity is % activity relative to the WSN (W) polymerase from at least 3 independent experiments. Inset square panels show typical gels. * and ** show statistical significance at p<0.05 and p<0.01, respectively, in a Student's t-test.

To determine which polymerase subunit(s) are required for the increased activity of the H5N1 polymerase in vitro, we measured polymerase activities derived from various artificial hybrid polymerases. In these experiments, the hybrid trimeric complexes consisted of one polymerase subunit from one or other of the H5N1 polymerases and the other two subunits from either the H1N1 or the H3N2 polymerases. In all cases the 3 polymerase subunits (PB1, PB2 and PA) were visualized on 7.5% SDS-PAGE, showing that these hybrids could form a functional complex (results not shown). Figure 2A and B show the results from hybrids of HK (H) with WSN (W). In both the ApG-primed (Figure 2A) and globin mRNA-primed transcription assays (Figure 2B), the hybrid formed from the PB1 subunit of HK showed no significant difference in activity from a WSN wild-type trimeric complex (Figure 2A and B compare lanes 1 and 3). By contrast, polymerase activities were significantly higher than wild-type WSN in the hybrid with the PA subunit of HK (Figure 2A and B, compare lanes 1 and 2). The mean polymerase activity was also increased in hybrids with the PB2 subunit of HK (Figure 2A and B, lane 4), although there was no statistically significant difference between these hybrids and the wild-type used (lane1). Overall, Figure 2A and B suggested that PA and possibly the PB2 subunits of HK may have contributed to the increased activity of the wild-type H5N1 polymerase seen in Figure 1B–E. It should be noted that position 627 of the PB2 subunit – known to be involved in host restriction [41], of HK is E (avian-like) whereas that of VN is K (human-like) - see Discussion.

To test if the results were specific to the HK-WSN hybrids tested above or were more generally valid, we constructed a set of different hybrid polymerases derived from the VN (H5N1) strain (V) in a background of the NT (H3N2) strain (N). We then compared the polymerase activity of these hybrids with the wild-type NT strain in vitro (Figure 2C and D). In these polymerase hybrids, only the PA subunit of VN increased activity compared to the wild-type NT in both the ApG-primed transcription assay (Figure 2C, compare lanes 1 and 2) and the globin-primed assay (Figure 2D, compare lanes 1 and 2). There was no increase in activity with the PB2 subunit (Figure 2C and D). In fact, there was a slight and statistically significant decrease in activity in hybrids containing the PB1 and PB2 subunits of VN in the ApG-primed transcription assay (Figure 2C, compare lanes 3 and 4 with lane 1). Taking the results of Figure 2A–D together, we conclude that the PA subunit of the two H5N1 strains (HK and VN) tested has a major influence in increasing the in vitro activity (Figure 1) of the H5N1 polymerase, when compared to classical H1N1 or H3N2 strains (Figure 2A and B, lane 4), although the PB2 subunit of HK may also have an effect - see Discussion.

Finally, to confirm the significance of the PA subunit of the H5N1 strains as a determinant of polymerase activity in vitro, we measured the activities of hybrids with PA derived from NT (H3N2), HK (H5N1) and VN (H5N1) in a complex with the PB1 and PB2 subunits derived from WSN (H1N1). Figure 2E (ApG-primed transcription) and Figure 2F (globin-primed transcription) showed that both HK PA (Figure 2E and F, lane 3) and VN PA (Figure 2E and F, lane 4) increased activity, whereas the PA subunit of NT decreased activity (Figure 2E and F, lane 2) compared to the wild-type WSN (lane 1). These results confirmed that PA subunits of both HK and VN H5N1 strains were responsible for increased polymerase activity in vitro. Thus the effect of PA on polymerase activity seems to be shared by at least these two H5N1 strains.

### The PA subunit of the H5N1 strains tested enhanced promoter binding

**Figure 3 pone-0005473-g003:**
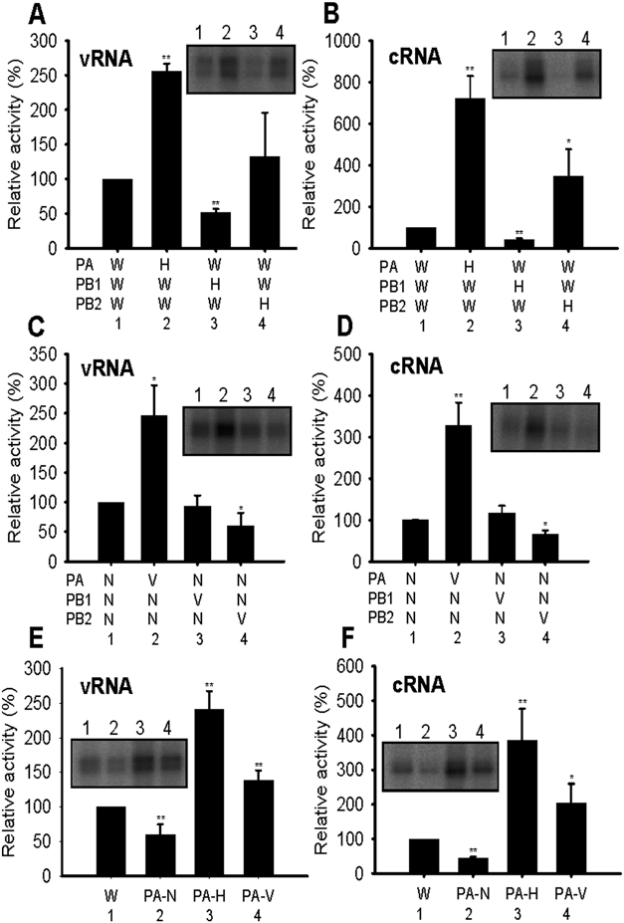
UV cross-linking of model vRNA and cRNA promoters to hybrid polymerases. Purified and quantified polymerases were incubated, in (A), (C) and (E), with ^32^P-labelled 3′ strand of the vRNA promoter in the presence of the unlabelled 5′ strand of the vRNA promoter, or in (B), (D) and (F), with ^32^P -labelled 3′ strand of the cRNA promoter in the presence of the unlabelled 5′ strand of the cRNA promoter. (A), (B) Hybrids between HK (H) and WSN (W), as indicated. (C), (D) Hybrids between VN (V) and NT (N), as indicated. (E), (F) WSN (W) polymerase was compared to hybrid polymerases of the PA of NT (PA-N), HK (PA-H) or VN PA (PA-V) with PB1 and PB2 from WSN. Relative activity is % relative to WSN from at least 3 independent experiments. Inset square panels show typical gels. * and ** indicate statistically significant differences (from W or N) at p<0.05 and p<0.01, respectively, in a Student's t-test.

Because the increased polymerase activity of H5N1 viruses (Figure 1) was observed in 4 independent assays, this suggested that a shared property of these assays was likely to mediate the effects. The replication and transcription assays of Figure 1 all depend on promoter binding prior to RNA polymerization suggesting that H5N1-derived polymerase might have enhanced promoter binding. To test this hypothesis, binding of radiolabelled promoter to various hybrid polymerases was assayed by UV cross-linking (see Materials and Methods). The PA subunit of HK in a WSN background enhanced binding to the model vRNA promoter (Figure 3A) and both PA and PB2 subunits enhanced binding to the model cRNA promoter (Figure 3B) when compared with wild-type WSN, but PA showed the greater effect (2.5–7 fold increase). In the case of the V-N hybrids, however, only the PA subunit of VN increased promoter binding by 2.5–3 fold (Figure 3C and D, compare lanes 1 and 2). To test whether the PA subunit of H5N1 viruses is generally required to increase promoter binding, we measured promoter binding of hybrids of all PA subunits tested in a WSN backbone. Both HK PA (Figure 3E and F, lane 3) and VN PA (Figure 3E and F, lane 4) significantly increased vRNA (Figure 3C) and cRNA (Figure 3D) promoter binding. The higher promoter binding activity of the PA-H hybrid compared to the PA-V hybrid (Figure 3E and F) is consistent with the higher activity of the HK compared to VT polymerase in Figure 1. Overall, these promoter binding results were in agreement with the in vitro transcription and replication assays (Figure 1 and 2), suggesting that the increased polymerase activity of the HK or VN H5N1 strains in vitro resulted from enhanced promoter binding - see Discussion.

### Dissection of regions within the PA subunit of A/Hong Kong/156/97 (HK) important for enhanced polymerase activity

**Figure 4:**
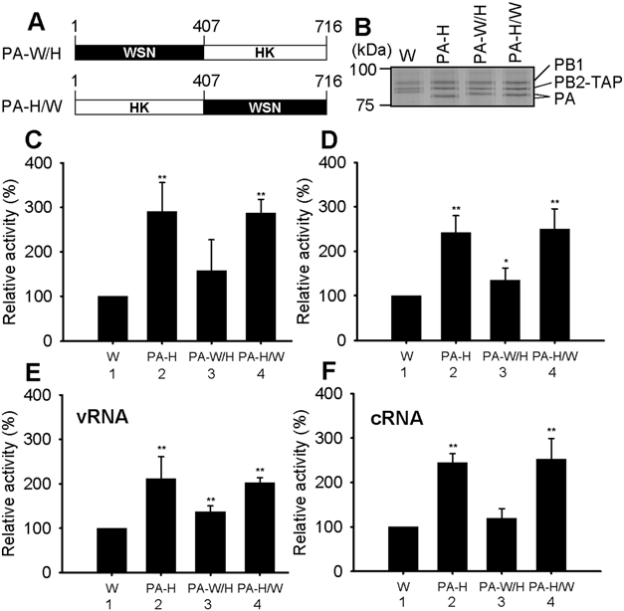
Polymerase activity and UV cross-linking of hybrids containing PA chimeras. (A) Design of PA chimeras. PA-W/H contained the N-terminal amino acids, 1–407 of WSN and C-terminal 408–716 of HK. PA-H/W contained the N-terminal amino acids, 1–407 of HK and C-terminal 408–716 of WSN. (B) 7.5% SDS-PAGE of WSN (W) polymerase and 3 hybrid polymerases with the PA subunit derived from HK (PA-H) or from chimeras, PA-W/H and PA-H/W, analysed by silver staining. The positions of PB1, PB2-TAP and the variable position of the PA subunits are shown. (C) ApG-primed transcription activity. (D) Globin mRNA-primed transcription activity. (E), (F) Promoter binding activity analyzed by UV cross-linking with the model vRNA promoter (in E) or the cRNA promoter (in F). Relative activity is % relative to WSN (W) from 4 independent experiments. * and ** indicate statistically significant differences from W at p<0.05 and p<0.01, respectively, in a Student's t-test.

In order to localize regions on the PA subunit, which might control the increased activity of the H5N1 polymerase, chimeras of HK PA and WSN PA (PA-W/H and PA-H/W) were constructed, dividing PA roughly in half at position 407 (Figure 4A). For these experiments, HK PA and WSN PA were selected, because the HK PA subunit showed the highest activity in all polymerase assays (Figure 1B–E) and because the PA subunit of WSN is an extensively studied classical strain [6], [17], [23], [46]–[47]. Figure 4B shows the variation in migration of the PA subunits of WSN (W), HK (see PA-H), and the 2 chimeras (WSN/HK (PA-W/H) and HK/WSN (PA-H/W)). Migration of the PA of both PA-W/H and PA-H/W chimeras was intermediate between the PA subunits of wild-type WSN (W) and wild-type HK (see PA-H). The polymerase activities were increased with the PA-H/W chimeras, compared to wild-type WSN, in both the ApG-primed transcription assay (Figure 4C, lane 4) and the globin mRNA-primed transcription assay (Figure 4D, lane 4), whereas the slight increase in activity with the PA-W/H chimera (Figure 4D, lane 3) was only marginally significant. In the promoter binding assay with either the model vRNA (Figure 4E) or cRNA promoters (Figure 4F), the HK/WSN (PA-H/W) chimera also showed strong promoter binding (Figure 4E and F, lane 4) comparable to the wild-type HK level (Figure 4E and F, lane 2). These results suggested that the N-terminal 1–407 amino acid of the PA subunit of HK made a large contribution to the increased polymerase activity by increasing promoter binding. There may also be a smaller contribution of the C-terminal region of the PA subunit of HK to vRNA promoter binding, but this is less significant than the N-terminal region (Figure 4E, lane 3).

### Ribonucleoprotein (RNP) reconstitution of polymerase activity in avian DF1 cells and human 293T cells in vivo

**Figure 5:**
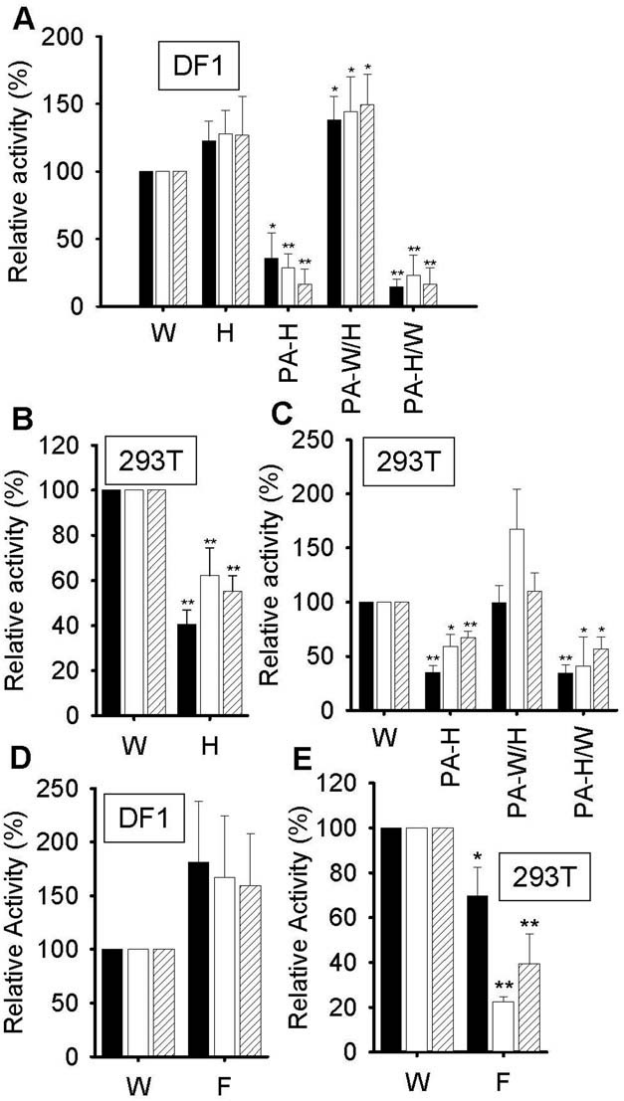
RNP reconstitution assays in chicken DF1 cells and human 293T cells. mRNA, cRNA and vRNA levels were measured by primer extension. (A) RNA levels of RNP derived from WSN (W), HK (H), or from the (PA-H) hybrid of WSN, or from 2 hybrids (PA-W/H or PA-H/W) containing the PA chimeras, were compared in DF1 cells. (B) RNA levels of RNP derived from WSN (W) and HK (H) were compared in human 293T cells. (C) RNA levels of RNP derived from PA-H, PA-W/H and PA-H/W were compared in 293T cells. (D) RNA levels of RNP derived from WSN (W) and Fujian (F) in DF1 cells. (E) RNA levels of RNP derived from WSN (W) and Fujian (F) in 293T cells. In all experiments, activities are expressed as a % relative to the wild-type WSN (W) from 3 independent experiments. Black, white and oblique lined columns show steady-state levels of mRNA, cRNA and vRNA, respectively. * and ** show statistically significant differences from wild-type WSN (W) at p<0.05 and p<0.01 in a Student's t-test. WSN NP was used for WSN and HK strains, and FJ NP was used for the FJ strain.

The in vitro assays described above would suggest that the N-terminal region of the PA subunit of HK was responsible for increased polymerase activity of HK polymerase mediated by enhanced promoter binding. In order to test if similar results were valid in vivo, RNP reconstitution assays [6], [17], [23] were performed in avian DF1 and human 293T cells and the results were compared. We initially tested the activity of wild type HK and WSN polymerase reconstituted as RNP in avian DF1 (chicken fibroblast) cells by expressing PB1, PB2, PA and NP with a vRNA neuraminidase reporter (see Materials and Methods) and assayed neuraminidase vRNA, cRNA and mRNA levels by primer extension (Figure 5A). For expression in DF1 cells it was necessary to use a reporter with a chicken Pol I promoter [48]. In DF1 cells all 3 steady-state neuraminidase RNA levels (vRNA, cRNA and mRNA) of the HK strain were similar to the WSN control (Figure 5A, compare W and H), whereas in 293T cells the levels of vRNA, cRNA and mRNA were all significantly less than that of the WSN strain (Figure 5B, compare W and H). However, RNP activity of the HK strain was approximately 50% of the WSN activity (i.e. it was not completely restricted) in 293T cells despite the presence of an avian-type Glu residue at position 627 of PB2 in the HK strain, consistent with previous replication studies with RNP of this same HK strain [49]. These results suggested that the HK strain did not show a significantly enhanced replication and transcription activity in vivo, in either chicken DF1 or human 293T cells, that might have been predicted from our in vitro results.

To test if another H5N1 strain, derived from ducks, showed similar trends to the HK H5N1 strain on reconstitution of RNP in chicken DF1 or human 293T cells, we reconstituted RNP from A/duck/Fujian/01/02 (H5N1) (FJ) with a neuraminidase vRNA reporter in either chicken DF1 or human 293T cells. In chicken DF1 cells that the mean steady-state levels of neuraminidase vRNA, cRNA and mRNA of FJ were all about 50% higher than the classical human WSN isolate (Figure 5D, compare W and FJ), although these differences was statistically significant only at the 90% significance level (mRNA, p = 0.05; vRNA, p = 0.09; cRNA, p = 0.10). In contrast, the FJ strain showed significantly lower levels of all 3 RNAs in 293T cells when compared with WSN (Figure 5E, compare W and FJ). Thus reconstituted RNP of an authentic avian strain of H5N1 had very similar properties, in both DF1 cells and 293T cells, to the HK strain isolated from humans (compare Figure 5A and D; compare Figure 5B and E).

Next, we tested the effect of the HK PA subunit alone in a hybrid (PA-H) where the other polymerase subunits, PB1 and PB2, were derived from WSN. This clearly showed that PA was inhibitory to replication and transcription, compared to wild-type, in both chicken DF1 and human 293T cells (Figure 5A and C). By testing the previously constructed PA chimeras (Figure 4A), we found that the N-terminal 1–407 region of PA (chimera PA-H/W) (Figure 5A and C) was responsible for the decreased activity in both cell types, although chimeras with the C-terminal half of PA (PA-W/H) showed slightly increased activity in DF1 (Figure 5A), but not in 293T cells (Figure 5B). Overall, these results showed that the N-terminal region (1–407) of the HK PA subunit (PA-H/W), when expressed as a RNP with PB1, PB2 and NP derived from WSN, decreased transcription and/or replication activity in both DF1 and 293T cells. This result suggested that the enhanced promoter binding mediated by the N-terminal region of PA of HK observed in vitro was detrimental to transcription and/or replication in vivo, at least in DF1 and 293T cells.

## Discussion

The aim of this study was to compare the RNA polymerase of 2 human-isolated avian H5N1 strains both with one another and also with 2 classical human isolates of differing subtypes i.e. A/WSN/33 (H1N1) and A/NT/60/68 (H3N2). One of the human-isolated avian H5N1 strains was A/Hong Kong/156/97 isolated in 1997, the other A/Vietnam/1194/04, isolated from Vietnam in 2004. These two strains were selected because the 1997 stain was the index strain - the first human isolate of H5N1 [50] while the 2004 Vietnam strain was a well characterized, genetically distinct, antigenic variant [51]. Moreover, A/Vietnam/1194/04 had acquired the PB2 627K host range mutation generally characteristic of mammalian viruses, whereas A/Hong Kong/156/97 retained PB2 627E characteristic of avian viruses [41]. After cloning the polymerase genes from both H5N1 strains, we expressed and partially purified recombinant, heterotrimeric (PB1, PB2 and PA) polymerase in 293T cells. Unexpectedly, we found that the polymerase of both the HK and VN H5N1 strains had significantly higher transcription and replication activities in vitro than the 2 classical human H1N1 and H3N2 subtypes (Figure 1).

To investigate which subunit, or subunits, of the polymerase was required for the increased activity in the H5N1 strains we constructed inter-strain hybrids where we mixed and matched subunits. Surprisingly, the only polymerase subunit in such hybrids to show increased activity in both H5N1 polymerases, irrespective of whether WSN or NT was used as a background, was the PA subunit (Figure 2). Significantly, we could convincingly attribute the increased activity of the H5N1 derived polymerase, compared to the WSN (H1N1) or NT-derived (H3N2) polymerase, to increased binding to both a model vRNA and a cRNA promoter (Figure 3).

The effect of the PB2 subunit, which had different amino-acids at position 627 and varied in sequence by 4% between the HK and VN strains, was apparently strain-specific, i.e. different results were obtained with the HK and VT PB2 subunits (Figure 2 and 3). Surprisingly, the HK PB2 seemed to promote activity more than the VT PB2, even although the VT strain has the PB2 627K residue characteristic of human-adapted strains, whereas HK has an E residue at this position characteristic of avian viruses. Our results may reflect the unusually active replication activity of this HK strain (see below). Further studies, however, are needed to confirm the different properties of the PB2 subunit of the HK and VN strains in our experiments, because the results might simply be a reflection of the different background strains (WSN or NT) used in the hybrids (Figure 2).

We subsequently focused on the PA subunit of HK because its polymerase was the more active of the 2 H5N1 strains studied. Analysis of chimeras between the N and C-terminal regions of the PA subunit of the HK and WSN strains suggested that the N-terminal region of the PA of the HK stain was the main determinant of the increased activity in vitro (Figure 4). Interestingly, it has been shown recently that the N-terminal region of the PA subunit (1–197 a.a.) has an endonuclease active site [13]. This observation is consistent with the involvement of the N-terminal region of PA in promoter binding, as demonstrated here. There was, however, a smaller effect with the chimera containing the C-terminal region of the HK H5N1 strain suggesting that the C-terminal region of PA may also be involved in increasing polymerase activity, but to a more limited extent. Subsequent attempts to characterize which amino acid(s) in the N-terminal region of the PA subunit of the H5N1 strains were responsible for the increased polymerase activity were inconclusive. We found that P28, R57 and S65 - all 3 amino acids characteristic of the PA subunit of both HK and VN, when introduced into the PA subunit of WSN, increased ApG-primed transcription activity in vitro, but this stimulation was not confirmed in other in vitro assays, e.g. globin-primed transcription, ApG synthesis (results not shown). Because S65 of the HK PA subunit encompassed a potential CKII phosphorylation site [52] we also tested for phosphorylation of HK PA, after in-gel trypsin digestion, by LC-MS/MS mass spectrometry. Only the non-phosphorylated peptide, GESIIVESGDPNALLK, was detected suggesting that phosphorylation had not occurred at this position 65 (underlined).

In order to test the effect of the PA subunit of the HK (H5N1) strain in vivo, the activity of the RNA polymerase of HK was studied in RNP reconstitution assays in both human 293T cells and avian DF1 cells. A reduced activity of the HK polymerase, which has a PB2 subunit with 627E, compared to WSN polymerase, which has a PB2 subunit with 627K, in 293T cells was initially expected. This expectation was based on the fact that the HK polymerase is essentially an avian isolate and would be expected to show host-restriction in human cells [41]. However, recent studies [49] on the same, HK polymerase show that it retains significant replication activity in 293T cells irrespective of whether PB2 at position 627 is E (avian-like) or K (human-like) residue. This suggested that the reduced activity of the HK RNP, when compared with WSN, in 293T cells (Figure 5B) is not caused by the 627E residue in PB2. Nevertheless, our results (Figure 5A) showed that the HK strain failed to significantly enhance polymerase activity in DF1 cells (although there was a slight increase in the mean value), as might have been expected from the increased promoter binding in vitro (Figure 3).

Further insight into the properties of the PA subunit of HK was obtained by studying the effects of a hybrid (PA-H) of the PA subunit of HK with the PB1 and PB2 subunits derived from WSN in RNP reconstitution assays. In both DF1 and 293T cells, activity of this hybrid was significantly reduced (Figure 5A and C). Confirming this and providing further information, we found that chimeras between the N-terminal region of the PA subunit of HK with the C-terminal region derived from WSN (PA-H/W) also markedly inhibited replication in both DF1 and 293T cells (Figure 5A and C). Consistent with this finding, no inhibition was observed with the reciprocal chimera (PA-W/H). This strongly suggested that the N-terminal region of the PA of HK mediated the reduction in RNP activity.

Although the N-terminal region of the PA of HK clearly influences polymerase activity both in vitro and in vivo, the question arises how this region stimulated polymerase activity in vitro (Figures 1–4) yet inhibited activity in the particular in vivo assays (Figure 5) used here? Binding of the influenza RNA polymerase to the proposed promoter corkscrew structure, formed by the 5′ and 3′ ends of all influenza RNA segments, is an initial step obligatorily required for polymerase activity whether this occurs in vitro or in vivo. Promoters thus define the site of initiation, yet the polymerase must release (promoter clearance) from the promoter because the promoter itself is transcribed. Subsequently the initial transcript is elongated to form short transcripts in the case of the in vitro assays (Figures 1–4) or longer transcripts similar to those produced in vivo in our RNP reconstitution assay (Figure 5). Differences between the in vivo and in vitro assays suggest a possible explanation for the different results obtained. The in vivo assay uses polymerase reconstituted as RNP and requires promoter binding followed by promoter clearance to allow initiation of transcription/replication and efficient synthesis of long RNA transcripts, without polymerase “stalling” (or polymerase release) from the template causing abortive transcripts. By contrast, the in vitro assays use free polymerase and promoter binding and only very limited promoter clearance, since only between 1 and 14 nucleotides are synthesized in the various assays used here (see Materials and Methods). It follows that a promoter that binds the polymerase efficiently in vitro, might not necessarily enhance transcription and replication in vivo, because promoter clearance might be impaired. In vivo, a strong promoter could result in excessive ‘pausing’ of transcription and replication leading to abortive initiation. Lower levels of influenza-specific transcripts would then accumulate.

Another potential reason for differences between the in vivo and in vitro assays is that non-viral, host factors, such as Pol II, MCM and hCLE - all factors proposed to interact with the influenza polymerase, might influence replication of ribonucleoprotein of HK and WSN to different extents in vivo, whereas these factors would be expected to be absent in the in vitro polymerase preparations [53]–[55]. Finally, the 293T and DF1 cells used here, might not reflect the properties of the virus during infection of the respiratory tract in humans. Thus, tissue-specific host factors, differing between normal cells (e.g. lung alveolar epithelia) of the respiratory tract and the 293T and DF1 cells used here, might significantly alter viral transcription and/or replication.

It might be argued that the differences observed here between the in vitro results and the in vivo results are not representative of H5N1 strains in general. However we have found that recombinant polymerase derived from an H5N1 duck strain (A/duck/Fujian/01/02) showed similar enhanced activity in vitro in the 4 different assays reported in Figure 1 (results not shown). Moreover in RNP reconstitution experiments in DF1 and 293T cells in vivo, this authentic avian strain has similar properties to the human-derived HK strain studied in detail here (see Figure 5D and E). Thus it is likely that our results - emphasizing the role of the N-terminal region of PA, are representative of H5N1 strains in general.

It is known that the passage history of A/HongKong/156/97 virus influences its virulence in mice [56]. A/HongKong/156/97 virus, used as a source of RNA for cloning here, had been passaged in eggs - a procedure known to attenuate virulence, so it is conceivable that the PB1, PB2 and PA clones isolated here derived from attenuated viruses. However, a comparison of the sequences of our PB1, PB2 and PA clones showed an imperfect correlation with mutations correlating with attenuation [56]. Thus PA had the amino acid (gly) at residue 631 correlating with the high pathogenic sequence. PB2 had the amino acid (asp) at residue 701 characteristic of the less pathogenic form. PB1 had 2 mutations (at amino-acids residues 17 (ala) and 456 (tyr) characteristic of the less pathogenic form) but a third at residue 711 (ser) was characteristic of the high pathogenic form. Given that the correlation of nucleotide mutations in the polymerase genes with pathogenicity was not investigated in detail [56], it is premature to conclude that the results reported here were influenced by passage history of the virus, although this still remains a possibility.

Thus we speculate that the N-terminal domain of the PA subunit of the HK strain identified here will have a role in virulence of H5N1 viruses, although this hypothesis would have to be directly tested by constructing recombinant viruses by reverse genetics and testing such viruses in mouse or ferret animal models. Although the importance of PB2 in virulence is not in doubt [40]–[41], it is becoming increasingly clear that virulence is multigenic and the role of PA cannot be ignored. Thus, recently, amino acid 515 of PA has been implicated in the virulence of an avian H5N1 strain in ducks [43]. The PA subunit was also proposed to act synergistically with the PB2 subunit in regulating activity of the A/Hong Kong/156/97 RNP complex in human cells in culture [49]. Moreover an interaction between PB2 and PA was proposed based on early studies of genetic suppression in temperature-sensitive, influenza vaccines [57].

In summary, a major aim of this study was to extend previous work showing that the N-terminal region of PA in influenza A/WSN/33 was multifunctional, and was involved in endonuclease activity, cap binding and promoter binding [6], [22]–[23], to H5N1 viruses. Here we have extended the previous studies of the PA subunit of the RNA polymerase of influenza A/WSN/33 (H1N1) to 2 more recently isolated strains, A/HongKong/156/97 (H5N1) and A/Vietnam/1194/04 (H5N1), and have shown that both these H5N1 strains possess dramatically higher polymerase activity in vitro, as a result of enhanced promoter binding, than those of the classical human strains (H1N1 and H3N2) tested. This enhanced activity, in the case A/HongKong/156/97, was shown to be mainly a function of the N-terminal region of the PA subunit. Overall, our data confirmed the importance of the PA subunit of 2 recent H5N1 strains in influencing promoter binding of the RNA polymerase. Potentially this enhanced promoter binding may be a factor in the virulence of H5N1 viruses, although this hypothesis remains to be tested in animal models.

## Materials and Methods

### Strains

RNA or cDNA clones isolated from the following influenza strains were used: A/HongKong/156/97 (H5N1), A/Vietnam/1194/04 (H5N1), A/duck/Fujian/01/02, A/WSN/33(H1N1) and A/NT/60/68 (H3N2). Position 627 of the PB2 subunit in A/HongKong/156/97 and A/duck/Fujian/01/02 is glutamic acid (E); the PB2 subunits of the other strains have a lysine (K) at 627.

### Plasmids

PB1, PB2, PA and NP-expressing plasmids of influenza virus A/WSN/33 (H1N1) pcDNA-PB1, pcDNA-PB2, pcDNA-PA, pcDNA NP, pcDNA-PB2-TAP and pcDNA-PA-TAP) have been described [17], [47], [58]. Full-length A/NT/60/68 (H3N2) PB1, PB2 and PA sequences were PCR amplified from the pBR322 clones, A/NT/60/68/2/62, A/NT/60/68/1 and A/NT/60/68/3/11 [accession numbers: J02138 (PB1), J02139 (PA) and J02140 (PB2)] [59]–[61] and inserted into pcDNA3A [45] using KpnI and NotI restriction sites generating pcDNA/60/PB1, pcDNA/60/PA, pcDNA/60/PB2, pcDNA/60/PA-TAP and pcDNA/60/PB2-TAP expression vectors. To construct expression vectors for A/duck/Fujian/01/02 PB1, PB2 and PA and NP [accession numbers: AY585483 (PB1), AY585504 (PB2) and AY5854625 (PA) and AY585420 (NP)] [62], full-length sequences were PCR amplified from pBD clones [63]. PB1, PA and NP PCR products were inserted into pcDNA3A using KpnI and NotI restriction sites generating pcDNA-FJ/01/02-PB1, pcDNA-FJ/01/02-PA and pcDNA-FJ/01/02-NP. PB2 was inserted into pcDNA3A and pcDNA-PB2-TAP using HindIII and NotI restriction sites generating pcDNA-FJ/01/02-PB2 and pcDNA-FJ/01/02-PB2-TAP, respectively.

To construct PB1, PB2 and PA expression vectors of A/HongKong/156/97(H5N1) [accession numbers: AF036362 (PB1), AF046095 (PA) and AF046093 (PB2)] [32], [49] and A/Vietnam/1194/04(H5N1) [accession numbers: AY651664 (PB1), AY651610 (PA) and AY651718 (PB2)] [64], RT-PCR was performed with Superscript II reverse transcriptase (Invitrogen) and Fusion polymerase (Stratagene) with RNA isolated from virus grown in embryonated chicken eggs. PCR fragments were inserted into pCR2.1-TOPO (Invitrogen) by TA cloning, and the coding region subcloned into pcDNA3A using KpnI and NotI sites, generating pcDNA/156/PB1, pcDNA/156/PA, pcDNA/156/PB2, pcDNA/156/PA-TAP, pcDNA/156/PB2-TAP, pcDNA/1194/PB1, pcDNA/1194/PA, pcDNA/1194/PB2, pcDNA/1194/PA-TAP and pcDNA/1194/PB2-TAP.

The A/Hong Kong/156/97 PB1 cDNA cloned here has 4 mutations at the following nucleotide positions (counting from the A of the initiator ATG as nucleotide 1) compared to the sequence of AF036362 (i) 318, G→A (ii) 355, A→G (iii) 1828, G→T (iv) 2276, A→G, causing 2 coding changes at amino acid residues 119, Met→Val and 610, Gly→Cys. PB2 and PA nucleotide and amino acid sequences of A/Hong Kong/156/97 were identical to the databases AF046093 (PB2) and AF046095 (PA). A/Vietnam/1194/04 PB2 cDNA cloned here also two coding changes (H60D and K189E) compared to the sequence of AY651718. The pPOLI-vNA plasmid has been described previously [17]. The PRC425.vNA plasmid was created by subcloning the EcoRI and BpuAI fragment of the A/WSN/33 NA gene of pPOLI NA into pPRC425 [48]. The plasmid contains part of the sequence of the NA gene under the control of a chicken Pol I promoter. To construct PA chimera plasmids, the N-terminal half or the C-terminal half of WSN PA was amplified by PCR and inserted into pcDNA/156/PA at KpnI and BamHI sites, or BamHI and NotI sites, generating pcDNA/WH/PA and pcDNA/HW/PA, respectively. Point mutations in the WSN PA gene were made by site directed mutagenesis [6], [17], [23] and were confirmed by full sequencing of the gene. Primer sequences are available upon request.

### Preparation of partially purified TAP-tagged polymerase

293T cells were transfected with the expression vectors containing PB1, PB2-TAP and PA subunit of each strain. Crude cell lysates were harvested 40 hours post-transfection and the polymerase were partially purified by the tandem affinity purification (TAP) method described previously [58]. The partially purified polymerase was analyzed by 7.5% SDS-PAGE with silver staining (Invitrogen) and quantitatively adjusted to a standard amount of polymerase [23].

### In vitro transcription assay

The quantitatively adjusted (see above), partially purified polymerase was used in the ApG-primed and globin mRNA-primed transcription assay as described previously [17]. Briefly, 1.5 µl of TAP-purified polymerase was mixed with either 10 ng/µl globin mRNA or 1 mM ApG as primer, 0.5 µM of the 5′ strand of the model vRNA promoter (5′ AGUAGAAACAGGCC 3′) (Dharmacon), 0.5 µM of the 3′ strand of the model vRNA (5′ GGCCUGCUUUUGCU 3′) (Dharmacon), 5 mM MgCl_2_, 1 mM DTT, 0.15 µM [α^32^P] GTP (3000 Ci/mmol, GE Healthcare), 1 mM ATP, 0.5 mM CTP and 2 U RNase inhibitor (Promega) in a reaction volume of 3 µl. After 60 min incubation at 30°C, transcription products were analyzed by 16% polyacrylamide gel containing 7 M urea in Tris-Borate-EDTA (TBE) buffer. Transcripts were detected by autoradiography and quantitated by phosphorimaging.

### In vitro replication assay

The dinucleotide initiation of replication assay was performed as described previously [6], [65]–[66], using adenosine instead of ATP. Briefly, 1.5 µl of adjusted polymerase was mixed with 0.02 µM [α^32^P] GTP (3000 Ci/mmol), 5 mM MgCl_2_, 1 mM DTT, 3 U RNase inhibitor, 1 mM adenosine and 1.75 µM each of the 5′ and 3′ strands of a model vRNA or model cRNA promoter in a 3 µl reaction volume. After 16 hours at 30°C, the ApG products were analyzed by 25% PAGE in 6 M urea in TBE buffer. ApG was detected by autoradiography and quantitated by phosphorimaging.

### UV cross-linking

UV cross-linking to model vRNA and cRNA promoters was performed as described previously [6], [17], [23]. Briefly, 2.5 µl of quantitatively adjusted polymerase [23] in the presence of 0.25 pmol (50,000 dpm) [γ^32^P]-labelled 3′ end of the vRNA promoter and 2 pmol of unlabelled 5′ end of vRNA promoter in a 5 µl reaction containing 10 mM HEPES (pH 7.5), 100 mM KCl, 2 mM MgCl_2_, 0.5 mM EGTA, 1 mM DTT, 10% glycerol and 8 U RNase inhibitor (Promega) was incubated at 30°C for 30 min. Reactions were then UV irradiated (254 nm) and the cross-linked products separated by 7.5% SDS-PAGE. Cross-linking was performed with the model cRNA promoter by replacing the [α^32^P]-labelled 3′ end of the vRNA promoter with approximately 0.25 pmol (50,000 dpm) [α^32^P]-labelled 3′ end of the cRNA promoter (5′ GGCCUUGUUUCUACU 3′) (Dharmacon) and replacing the unlabelled 5′ end of the vRNA promoter with 2 pmol of the unlabelled 5′ end of the cRNA promoter (5′ AGCAAAAGCAGGCC 3′) (Dharmacon). The products were detected by autoradiography and quantitated by phosphorimaging.

### RNA isolation and primer extension assay

293T cells were transfected with expression vectors of PB1, PB2 and PA subunit of each strains (WSN, NT, HK and VN), pcDNA-NP (WSN) and pPOLI-vNA (WSN). Subconfluent monolayers of DF1 cells in DMEM medium supplemented with 10% FCS, in 60 mm dishes were transfected with Lipofectamine 2000 reagent (Invitrogen) according to the manufacturer. Briefly, 2 µg each of pcDNA-PB1, pcDNA-PB2, pcDNA-NP, pPRC425.vNA plasmids and 2 µg of PA plasmid of each strains (WSN, HK or mutant) were diluted with 150 µl OPTI-MEM (Invitrogen). This solution was then mixed with 20 µl of Lipofectamine 2000 reagent (Invitrogen) previously diluted in 100 µl OPTI-MEM. 24 h. later total cell RNA was isolated using TRIzol reagent (Invitrogen). RNA was then analyzed in a primer extension assay using three primers-one for vRNA, one for mRNA and cRNA, one for 5S rRNA as an internal control [6], [17], [23]. Transcripts were visualized by 6% polyacrylamide gel containing 7 M urea in TBE buffer and quantitated by autoradiography and phosphorimaging.
